# Stress Management in Pre- and Postoperative Care Amongst Practitioners and Patients in Cardiac Catheterization Laboratory: A Study Protocol

**DOI:** 10.3389/fcvm.2022.830256

**Published:** 2022-07-01

**Authors:** Andrea Block, Klaus Bonaventura, Patricia Grahn, Felix Bestgen, Pia-Maria Wippert

**Affiliations:** ^1^Medical Sociology and Psychobiology, Department of Health and Physical Activity, University of Potsdam, Potsdam, Germany; ^2^Faculty of Health Sciences Brandenburg, Joint Faculty of the University of Potsdam, the Brandenburg Medical School Theodor Fontane and the Brandenburg University of Technology Cottbus – Senftenberg, Potsdam, Germany; ^3^Department of Cardiology and Angiology, Ernst von Bergmann Hospital, Potsdam, Germany

**Keywords:** stress management, mindfulness-based stress reduction, psychoeducation, standardized patient information, stress intervention, distress, study protocol, cardiac catheterization (CC)

## Abstract

**Background:**

As the number of cardiac diseases continuously increases within the last years in modern society, so does cardiac treatment, especially cardiac catheterization. The procedure of a cardiac catheterization is challenging for both patients and practitioners. Several potential stressors of psychological or physical nature can occur during the procedure. The objective of the study is to develop and implement a stress management intervention for both practitioners and patients that aims to reduce the psychological and physical strain of a cardiac catheterization.

**Methods:**

The clinical study (DRKS00026624) includes two randomized controlled intervention trials with parallel groups, for patients with elective cardiac catheterization and practitioners at the catheterization lab, in two clinic sites of the Ernst-von-Bergmann clinic network in Brandenburg, Germany. Both groups received different interventions for stress management. The intervention for patients comprises a psychoeducational video with different stress management technics and additional a standardized medical information about the cardiac catheterization examination. The control condition includes the in hospitals practiced medical patient education before the examination (usual care). Primary and secondary outcomes are measured by physiological parameters and validated questionnaires, the day before (M1) and after (M2) the cardiac catheterization and at a postal follow-up 6 months later (M3). It is expected that people with standardized information and psychoeducation show reduced complications during cardiac catheterization procedures, better pre- and post-operative wellbeing, regeneration, mood and lower stress levels over time. The intervention for practitioners includes a Mindfulness-based stress reduction program (MBSR) over 8 weeks supervised by an experienced MBSR practitioner directly at the clinic site and an operative guideline. It is expected that practitioners with intervention show improved perceived and chronic stress, occupational health, physical and mental function, higher effort-reward balance, regeneration and quality of life. Primary and secondary outcomes are measured by physiological parameters (heart rate variability, saliva cortisol) and validated questionnaires and will be assessed before (M1) and after (M2) the MBSR intervention and at a postal follow-up 6 months later (M3). Physiological biomarkers in practitioners will be assessed before (M1) and after intervention (M2) on two work days and a two days off. Intervention effects in both groups (practitioners and patients) will be evaluated separately using multivariate variance analysis.

**Discussion:**

This study evaluates the effectiveness of two stress management intervention programs for patients and practitioners within cardiac catheter laboratory. Study will disclose strains during a cardiac catheterization affecting both patients and practitioners. For practitioners it may contribute to improved working conditions and occupational safety, preservation of earning capacity, avoidance of participation restrictions and loss of performance. In both groups less anxiety, stress and complications before and during the procedures can be expected. The study may add knowledge how to eliminate stressful exposures and to contribute to more (psychological) security, less output losses and exhaustion during work. The evolved stress management guidelines, training manuals and the standardized patient education should be transferred into clinical routines.

## Introduction

Cardiovascular diseases and their treatment are steadily rising in modern society and cardiac catheterizations are considered as a low risk, routine diagnostic procedures in this realm (1). In 2014, ~885.000 cardiac catheterizations and 342.000 interventions were conducted in Germany. In 2019, 836.202 left heart cardiac examinations and 297.094 percutaneous catheter intervention (PCI) were examined, an increase of 3.3 and 0.5%, respectively, compared to the previous year (2). The rising numbers of examinations entailed an increased workload by a nearly constant number of practitioners. Consequently, the time of preparation and post processing for the cardiac catheterization examination decreases as well as the recovery time for the practitioners between the examinations. No control over workload and hectic work environment are two factors that were most strongly associated with distress and burnout in cardiologists (3, 4). Beside the tremendous impact of burnout for the individual physician, like greater risks for alcohol and substance abuse and suicide, it also negatively impacts patient care (4). Systematic reviews have shown that physicians' burnout are associated with suboptimal quality of care and patients safety (5). Stress overload and burnout in physicians may consequently result in medical errors and ultimately add to the ~19.000 dead patients very year due to treatment errors (6). Medical errors are often associated with feelings of guilt and shame that often adds further to distress and depressive states in physicians (7, 8). In addition, ‘fatigue due to long duty hours‘ and ‘having other things to take care of‘ were identified as two the most common self-reported reasons for medical errors (7).

Cardiac catheterizations are challenging for both practitioners and patients: there are physical and psychological stress factors that are afflicted with uncertainty and high demands during this procedure. But practitioners and patients are afflicted differently by different stressors. For practitioners several potential sources of stress had been identified during the cardiac catheterization examination. Physical stressors range from restricted respiration and dehydration to orthopedic strain, chronic work-related pain, increased body temperature and limited mobility caused by surgical masks and 5–8 kg heavy lead apron as a protection against radiation (6). Besides the physical stress, practitioners are also exposed to a high psychological demand. They have to be highly concentrated under time pressure and may be confronted with complications that can be life threatening for their patient. In other words, practitioners can be under physical and mental strain during a cardiac procedure that triggers the stress response and releases stress hormones. Sympathetic over parasympathetic activation is key feature of the stress response and can be measured by heart rate variability. In addition, increased stress leads to a reduced mental flexibility and capability which might have an impact on the result of the cardiac catheterization procedure (9). Thus, chronic stress does not only affect the health of cardiologists (10, 11), it also has an impact on the surgical performance and therefore on patients health and safety (12–14). Therefore, it seems almost inevitable to implement preventive interventions that are on the one hand successfully reduce perceived distress and tension in practitioners and on the other hand are efficient in time and costs and fit in to the daily routines of the clinic. Mindfulness-based intervention are evaluated to substantial lessen symptoms of stress, depression and anxiety and improve quality of life and physical capability (15).

On the other side of the surgical table, a cardiac catheterization is often an anxiety-provoking and inscrutable procedure for the patients as its concerns the examination of an essential organ, the heart (6, 16). Most patients appraise an impending cardiac catheterization as a threat of harm or challenge and experience psychological distress. Feelings of anxiety, loss of control, and fear for complications are often triggers of stress (17, 18). Unmanaged psychological distress and tension may lead to considerable complication during the cardiac catheterization, such as cardiac dysrhythmia, vessel spasm, and vessel laceration (19). Anxiety and uncertainty can also influence postoperative pain, pain medication and wellbeing (20). Therefore, it can be beneficial to reduce tension, anxiety and uncertainty with the aid of a psychoeducational intervention prior to the examination (21, 22). Studies have shown that stress-reducing interventions like music, therapeutic touch and massage have a desirable impact on physiological and psychological outcomes in patients undergoing cardiac examinations (23, 24). Preoperative cognitive and educative interventions were attested to positively influence the level of anxiety, wellbeing, treatment satisfaction, pain, and the amount of pain medication in the patient (25–30). It has been also shown that it is important to involve the patient into the perioperative pain management (21, 31). A sense of control and self-efficacy through psychoeducative interventions prior operations are associated with a faster reduction in post-operative pain and recovery (32, 33).

Although there is still only limited research on stress-reducing interventions in catheterization labs, a few studies on psychological preparation and video-based psycho-educative interventions in patients have shown beneficial results concerning pre- and perioperative distress, anxiety and coping as well as postoperative recovery and wellbeing (34–37). Less stressed and anxious patients may lead to less complications and reduced durations of cardiac catheterizations that could be beneficial for both patients and practitioners. A lower risk of complications and well-prepared patients may reduce the distress and pressure on the staff at the catheterization lab that in turn could be beneficial for patient safety and therapeutic outcome. To the best of our knowledge, there is no study protocol or study on stress-management in clinical settings that address both group—practitioners and patients—simultaneous. We expect a positive, bidirectional influence from both interventions to both intervention groups.

The objective of the study is to develop and implement an intervention for both practitioners and patients that aims to reduce the physical and psychological strain of a cardiac catheterization. This study tries to answer the following research questions:

- Whether and to what extent are cardiac catheterization interventions physically and psychological stressful for both patients and practitioners?
- Whether and to what extent does a standardized video-based patient education lead to less stress, anxiety, surgical complications, postoperative hospitalization, as well as better recovery, compliance and well-being?
- Whether and to what extent can practitioners benefit from operational guidelines at the catheterization lab and a stress management intervention in terms of reduced stress during work at the catheterization lab and a faster recovery after a workday?

## Methods and Analysis

### Study Design

**Figure 1:**
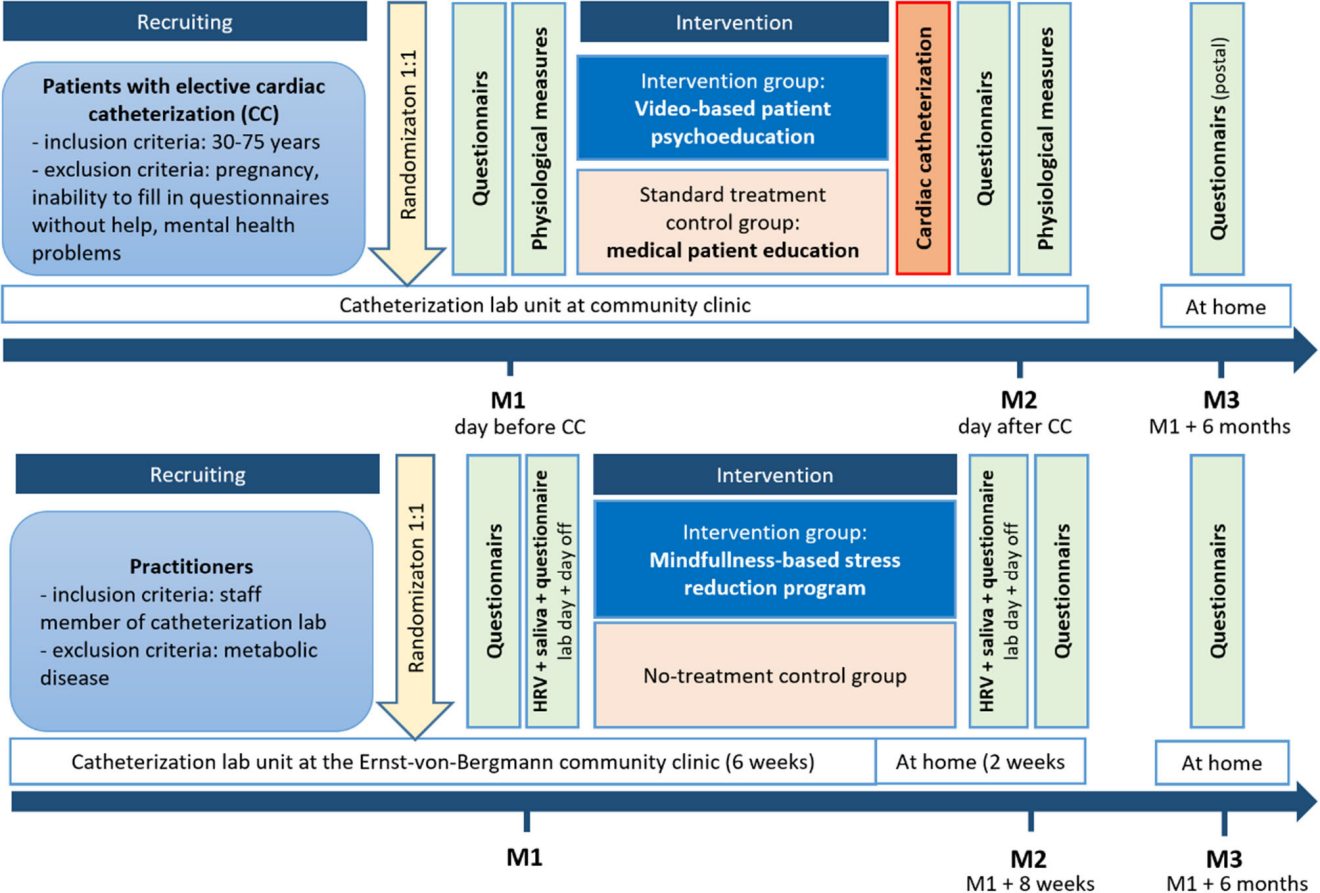
Randomized controlled intervention trials with parallel groups and three measurement points (before and after intervention, 6 months after baseline) for patients with elective cardiac catheterization and practitioners at the catheterization lab (before and after intervention, 6 months after baseline).

The multicenter study “Heartbeat” implement a randomized and controlled pre-post-study design involving two intervention trials with parallel groups, for patients with elective cardiac catheterization and practitioners at the catheterization lab, respectively (Figure 1, DRKS No: 00026624^1^). The patient study with a 1:1 randomization by person and a standard treatment control group involves three measurement points: the day before (M1 = admission day) and the day after the video-based stress management intervention and cardiac catheterization examination (M2 = CC examination day), and 6 months after baseline (M3). The MBSR intervention trail for the practitioners at the CC lab involves a 1:1 randomization by group and a no-treatment control group with three measurement points: before (M1) and after the 8-week (M3) intervention with a mindfulness-based stress reduction program, as well as 6 months after baseline (M3). The study takes place at the two clinic sites of the Ernst-von Bergmann community clinic, in Potsdam (patients and practitioners) and Bad Belzig (only patients). The time of data collection is estimated with ~1 h for all questionnaires and 2 days for the physiological measurements (practitioners). The data collection period is planned from 01/2021 to 12/2022.

### Participants

All patient with an elective cardiac catheterization, hospitalized in the Ernst-von-Bergmann clinic in Potsdam or Bad Belzig, and all staff members at catheterization lab at the Ernst-von-Bergmann clinic in Potsdam are eligible for participation. Patients between the ages of 30 and 75 years with a scheduled heart catheter examination were recruited for study participation. The following criteria should be met: the CC examination have to be elective and non-acute. In addition, only patients, scheduled for coronary angiography, are included. Exclusion criteria were: the inability to fill in a questionnaire, pregnancy and mental health problems. Only practitioners—physicians or nurses—that work at the catheterization lab are included in the intervention trail. The exclusion criterion for practitioners is the presence of an endocrinological metabolic disease. All participants have to sign informed consent after receiving written and verbal information. The study is conducted according to the Declaration of Helsinki (ethics approval 03/06/2021, ethics review board University of Potsdam No. 38/2021) and complied with the Consolidated Standards of Reporting Trials (CONSORT).

### Sample Size Determination

We calculated the sample size for two patient groups for two measurement points based on a pilot study. The sample was powered to assume a medium effect size (*d* = 0.25) and a minimum detectable clinical difference of 0.5 standard deviation on the (z-standardized) main criterion: perceived stress. For the main criterion, the power for significant group differences is 1-β = 0.93 with a case number of *N* = 60 people per group (38). As the sample for the practitioners was limited to all members of the catheterization lab of the clinic who fulfill the inclusion criteria, a sample size determination was not required.

### Recruitment, Screening and Informed Consent

The recruitment of the participants for both the patients and practitioners take place directly on site in the clinic by a research assistant of the study. Patients will be consecutively asked to participate on the day they arrive at the clinic, preconditioned they fulfill all inclusion criteria proofed by doctors. All practitioners of the catheterization lab from the Ernst-von-Bergman clinic are eligible for the study and will be recruited by the study personnel. All patients and practitioners will provide written, informed consent after a comprehensive information about the aim and the procedures the of study in written and verbal form. All participants will be additionally informed about their right to refuse to participate or to withdraw consent to participate at any time of the study (before anonymization of data) without reprisal.

### Randomization Procedure and Blinding

The process of randomization to the allocation arm was performed prior to recruitment via a randomization list. This randomization list was generated via the computerized randomization tool Research Randomizer (https://www.randomizer.org/). Participants will be randomly assigned to either the intervention or control condition according to the randomization list. Patients and practitioners cannot be blinded due to their active role in the intervention. Practitioners at the cardiac catheterization laboratory are blinded to the group allocation of the patients.

### Experimental Procedure/Intervention

As both patients with an elective cardiac catheterization (CC) examination and practitioners at the catheterization lab face different stressors, both groups received different interventions for stress management.

The CC for the included study participants is elective (non-acute) and medically recommended to evaluate or confirm coronary artery disease, congenital heart disease, heart failure, heart valve disease or aorta dysfunction, heart muscle function and to decide for further treatment. On the admission day (M1), all patients get the usual medical patient information from a cardiologist and anesthetist and get prepared for the examination (fasting, no smoking, evaluation of medication). All patients that fulfill the inclusion criteria concerning age and CC examination will be randomly dedicated to intervention group and control group and subsequently contacted by a research assistant who present the study and its aims and answer all questions. All participants have to sign informed consent after receiving written and verbal information. Afterwards all participants receive the questionnaires for M1 (pre-intervention questionnaire) and only patients in the intervention group get and only patients in the interventions group get the intervention (see The intervention for patients). The next day (M2), all patients get the CC examination at the catheterization lab. During the CC the patient is usually awake and conscious as the hostipal policy is not to administer any tranquilizer, sedatives and anxiolytics as a routine before or during CC procedure. However, patients get sedatives if needed and the type of sedative and dose is documented in a questionnaire by physicians or nurses of the CC lab. After disinfection and local anesthetization of the insertion site (groin, wrist or rarely crook of the arm), the catheter is inserted through a plastic introducer sheath. The catheter is guided through the blood vessel to the coronary arteries and coronary angiography is done by injecting a contrast dye that is visible in X-ray images. The following further diagnostic and interventional procedures can be performed during a cardiac catheterization: myocardial biopsy, levocardiography, right ventriculogram, coronary angiogram, and treatments for narrowing or blockages in the blood vessels like balloon dilatation and stent placement. The aftertreatment includes a bed rest for several hours in the ward until the following day of discharge. The participants fill in the post-intervention questionnaire during the bed rest/stay in the ward (M2).

*The intervention for patients* focusses on a video-based standardized patient information about the cardiac catheterization examination and further involves a short patient education with different stress management technics. The video-based standardized patient information gives information about the aim and general process of the CC, like location and aftercare of the puncture, position of the participants during the examination how the procedure is monitored. Further information comprises potential but rare complications and side effects like additional interventions, circulatory disruptions, cardiac arrhythmia, bleedings and (temporary) pain. The second part of the video broaches the issue of stress management technics to handle anxiety, tension and malaise. It provides brief instructions for relaxation, meditation and guided imagery techniques as well as cognitive distraction tasks, and cognitive reframing. All cognitive techniques are easy to apply after one presentation and involve methods to stop negative thoughts, distract from unhelpful thought patterns, to guide attention and positive expectations, to lower tension and support relaxation. For example, the patient should recite the alphabet and imagine a positive thing or event for every letter that he/her would be able to do after the CC. The patient should guide his/her attention to their most pleasant vacation and reactivate the feelings and thoughts present at that time. If a patient feels discomfort or pain, he/she should guide his attention to his/her big toe or left hand for cognitive distraction. The video encourages the patient to speak to the doctors and nurses about complaints and needed assistance. The video is shown to the patients the day before the CC via a tablet bedside in the clinic and is complementary to the non-standardized patient education by the medical doctor that is clinical routine for both intervention and control group.

*The intervention for practitioners* includes an 8-week Mindfulness-based stress reduction (MBSR) program based on the Jon Kabat-Zinn method (39, 40), which take place weekly for 1.5 h. For the first 6 weeks, the intervention is supervised by an experienced MBSR practitioner directly at the clinic site. The following 2 weeks, it takes place at home guided by an audio guide and an exercise book. The MBSR program includes body scan, sitting meditation and Hatha-yoga exercises, as well as breathing exercises throughout all three types of meditation. During the body scan every part of the body is systematically and conscious sensed in a lying position to enhance mindfulness. Key component of the sitting meditation is the upright position and conscious focus on breathing and staying in a mindful state. The different yoga poses (asanas) comprise balance and stretching elements for lying and upright positions. The exercise within MBSR differs from week to week with regard to the yoga elements, but every single routine starts with body scan and sitting meditation.

Additionally, an operative guideline for interaction with colleagues, conduct during and after complications and traumatic events during a cardiac catheterization examination was developed on basis qualitative interviews with staff members of the catheterization lab prior to the study. These guidelines for mindful and self-aware behaviors are printed and hang out as a poster at the catheterization lab.

### Strategies to Improve Adherence to Interventions

As the intervention video takes only 10 min to watch, adherences strategies for the patients focusses on improved questionnaire return and complete data. To ensure that all questionnaires are completed and returned, a close relationship and support between participant and study staff are stablished. After recruitment, study education and informed consent, participants are asked to fill in the questionnaire for baseline and return it to the staff member. For after-intervention questionnaire (M2) this routine is assured by the nurses at the catheterization ward. To secure the follow-up measurement (M3, 6 months after baseline), a study member contacts the participants via telephone call and sends the questionnaire by post with a prepaid envelope for the return. For the practitioners the adherence strategy focusses on a low-threshold and time-saving training intervention directly at clinic side. Furthermore, adherence problems and barriers are assessed in all follow-up questionnaire. The measurement of HRV on two lab and two leisure days (before and after intervention) is realized by self-applied chest strap heart monitor, a smartphone and an app for the HRV measurement, all provided by the study staff.

### Outcomes

The primary outcomes for patients are reduced complications and less sedative medication during CC (documented by nurses at the CC lab), and less tension/strain (physiological parameters like pulse, blood pressure, heart frequency), perceived stress (PSS) and better mood (POMS) measured by established and validated self-report questionnaires. Further secondary outcomes for patients are better pre- and post-operative wellbeing, chronic stress (TICS), depression and anxiety (HADS), panic disorder, psychosocial functioning (PHQ-9), life events (ILE), general health (SF-12), satisfaction with health and sleep (41). Additionally, patients are asked about their life style, socioeconomic status and satisfaction about the intervention. At the CC lab, nurses document the behavior of the patients during the cardiac catheterization (agitation, duration of hospital stay, tranquilizer/sedativa use, and level of information) and the number of complications occurred during the procedure. The primary outcomes for practitioners—perceived (PSS) and chronic stress (TICS)—are measured by established and validated self-report questionnaires. Additionally, stress load and recovery are assessed by physiological measurements: circadian profile of HRV (42–44), saliva cortisol (45), alpha-amylase (46) and lysozyme on two working/lab days and two days off as well as the following night, prior to and after the intervention, respectively. The HRV measurements base on beat-to-beat R-R intervals and comprises outcomes for the total activity of the autonomic nervous system [standard deviation of RR intervals (SDNN)], parasympathetic function [root mean sum of squared distance (RMSSD)] and stress load and recovery (number and percentage of R-R intervals lower or >50 msec). Saliva samples are taken on nine defined time points during the day (immediately after awakening, 30, 45, 90, 150 min after awakening, before lunch, 4 p.m., before dinner and going to bed) on a working/lab day and day off. Weight and mood (POMS) are assessed simultaneously as control variables. The practitioners are further asked about vital exhaustion (VE), anxiety and depression (HADS), tension, effort-reward imbalances (ERI), social support (BSSS), satisfaction with health and sleep (41) as well as life style, socioeconomic status, expectations and adherence (barriers) to the intervention program.

Details on outcomes, questionnaires and measurement points are provided in Table 1 for participants and Table 2 for practitioners.

**Table 1:** Outcome measurements for patients with elective cardiac catheterization.

**Outcome**	**Measurement**	**Assessment**		
		**M1** **(day before CC)**	**M2** **(day after CC)**	**M3** **(6 months after M1)**
Chronic stress	Trier inventory for chronic stress (TICS) (47)	X		X
Perceived stress	Perceived stress scale-−10 items (PSS-10) (48, 49)	X		X
Life events	Inventory of stressful life-events (ILE) (50)		X	
Depression	Patient health questionnaire-−9 items (PHQ-9) (51)	X		X
Anxiety and depression	Hospital anxiety and depression scale (HADS) (52, 53)	X	X	
Health-related quality of life	Short form health-−12 items (SF-12) (54)	X		X
Pain	Visual analog scale (VAS)	X	X	X
Mood	Profile of mood state (POMS) (55, 56)	X	X	
Satisfaction with health and sleep	Visual analog scale (VAS)	X	X	X
Life style factors	Physical activity, smoking, alcohol consumption, medication, critical life events	X		X
Sociodemographic characteristics	Sex, Age, education, marital status, job position, income	X		
Satisfaction with intervention			X	
Behavior while CC*				
Physiological parameter while CC*	Pulse, blood pressure, heart frequency	X	X	

*(additional) measurement during CC.

**Table 2:** Outcome measurements for practitioners at the catheterization lab.

**Outcome**	**Measurement**	**Assessment**		
		**M1** **(before intervention)**	**M2** **(after intervention)**	**M3** **(6 months after M1)**
Chronic stress	Trier inventory for chronic stress (TICS) (47)	X		X
Perceived stress	Perceived stress scale-−10 items (PSS-10) (48, 49)	X		X
Effort-reward imbalances	Effort-reward imbalances questionnaire (ERI) (57)	X		X
Anxiety and depression	Hospital anxiety and depression scale (HADS) (52, 53)	X	X	
Vital exhaustion	Maastricht vital exhaustion questionnaire (VE) (58)	X	X	
Tension	Visual analog scale (VAS)	X	X	X
Mood	Profile of mood state (POMS) (55, 56)	X	X	
Satisfaction with health and sleep	Visual analog scale (41)	X	X	X
Life style factors	Physical activity, smoking, alcohol consumption, medication, critical life events	X		X
Social support	Berlin social support scale (BSSS) (59)		X	
Sociodemographic characteristics	Sex, age, education, marital status, job position, income	X		
Expectation of/satisfaction with intervention		X	X	
Heart rate variability	Activity of the autonomic nervous system (SDNN), parasympathetic function (RMSSD)	X	X	
Stress profile	Cortisol, alpha-amylase (saliva samples) on nine defined time points during the day (two lab days and two days off)	X	X	
Weight	Scale	X	X	

### Data Management

The study is conducted in compliance with the EU's General Data Protection Regulation (GDPR) (60). All collected data will be processed pseudonymized. After completion of data collection all data will be anonymized for data analysis. Compliance with data protection is ensured by strictly anonymized data input into electronic data base. Personal data will be collected on the day of the recruitment by a member of the research group. This data will be assessed in written form and stored in a secured case with restricted access during the whole project.

### Statistical Analysis

All statistical analyses will be performed using SPSS. The data of the practitioners will be mainly analyzed descriptively due to the small sample size. Interferential statistics will be used to identify group differences. A comparison with norms of the German general population will be performed to assess the stress of the practitioners in the cardiac catheter laboratory. Baseline and post-interventional physiological data like circadian profile slopes of HRV and derived indicators (SDNN, RMSSD, number and percentage of R-R intervals lower or >50 msec) as well as saliva cortisol, alpha-amylase and lysozyme measurements will be compared on an intraindividual level. The data of the patients will be analyzed according to a multivariate variance analysis (between-subject factor: intervention group vs. control group). Sociodemographic data, sedative medication, adherence to the intervention will be implemented as control variables or stratification variables for subgroup analysis. Statistical significance is set at *p* < 0.05 (two-sided) and effect sizes will be reported.

## Discussion

The randomized control intervention study presented in this protocol will test the effectiveness of stress management interventions for both patients who undergo elective cardiac catheterization and practitioners who work in the catheterization lab. The intervention for patients comprises a video-based standardized information about the cardiac catheterization examination and a patient psychoeducation with stress management techniques like relaxation, meditation and guided imagery as well as cognitive distraction and reframing. The intervention video is presented at the admission day before the cardiac catheterization. The intervention for practitioners includes an 8 weeks Mindfulness-based stress reduction (MBSR) program with body scan, sitting meditation, Hatha-yoga and breathing exercises for 90 min per week at the clinic site and at home. Additionally, operative guidelines for mindful and self-aware behaviors during the catheterization procedure and in case of complications and adverse events are evolved and presented at the lab. Generally accepted and validated instruments and questionnaires are implemented to measure the effect of the intervention.

Although cardiac catheterizations are one of the most frequently used standard diagnostic procedures in invasive cardiology, there are only a few studies that analyse stress management interventions for practitioners at catheterization labs and only a few methodological reliable and up-to-date intervention studies that analyse video-based psychoeducative interventions (9). Given that burnout is reported by 50% of cardiologists and that this trend is driven by systemic demands and inefficiencies of the healthcare system (61), practitioners at catheterization labs seem to be a risk group of chronic stress and all health consequences that come along with it like sleep disorders, depression, cardiovascular diseases, obesity, Type-2 diabetes (62).

On this account we evolved a time effective and easy to implement stress management program for both practitioners at catheterization labs and patients that undergo cardiac catheterization. Negative health consequences for the practitioners and potential risks for patient safety may be best mitigated by stress-management interventions like the Mindfulness-based stress reduction program (MBSR). Randomized control studies proof that MSBR programs reduce depression, anxiety and stress (15) and improve chronical pain, relapse rate in depression as well as general health and relationships (63). As an easy to learn group program that focuses upon mindfulness meditation, body scanning and simple yoga postures, the mindfulness-based stress reduction program is a low-threshold intervention that could be directly implement at the clinic site. The MBSR program can be easily applied in daily routines and as it is equipment independent and transferable to other locations like home. A lack of adherence to the intervention program is an anticipated hazard for the study results. The intervention comprises a two-step program with a 6-week supervised MBSR module directly at the clinic site followed by a participant-led intervention at home for 2 weeks supported by an audio guide and an exercise book. Adherence problems are assessed by open-ended questions to the participants in all follow-up questionnaires. This information will be used to identify a possible shift in adherence and usual barriers that have to be addressed in transfer recommendations for practice and clinical routine. Concerning patients at the catheterization lab, there are evidence that standardized, video-based patient information (64–67) and relaxing interventions (65) prior to invasive operational examinations have positive effects on patient anxiety, tension, general wellbeing and perioperative pain management (21, 28). Preoperative information and perioperative stress coping seem to increase self-efficacy, controllability as well as satisfaction with the CC examination (64, 65, 68, 69). Nevertheless, there are meta-analysis and intervention studies that revealed how pre-surgical expectations of patients affect post-examination outcomes like length of stay, post-surgical complications and recovery (70). Negative expectations and previous experiences may trigger potential nocebo-related effects and could have an effect on pain perception and perceived stress (71, 72). Our stress-management intervention for patients addresses expectations toward the CC examination and give instructions to get awareness of negative expectations and promote positive expectation. As we do not assess expectations in our control group this could still be a threat to validity of our study results or a limitation in our study.

Both stress-management intervention for patients and practitioners are expected to have beneficial effects on the involved individuals. We expect to significantly minimize tension, perceived stress during the cardiac catheterization in patients of the intervention group. Additionally, we expect a favorable effect on physiological outcome like heart rate, blood pressure (in patients) and heart rate variability (in practitioners). An effective stress reduction should be favorable for the individual patients but it should further prevent complications and stressful incidents during the cardiac catheterization. On this account a successful patient intervention may add to less hazard and more safety in the workplace, improved working conditions and occupational safety, preservation of earning capacity, avoidance of participation restrictions and loss of performance for the practitioners. These positive aspects of work safety for the staff members of the catheterization lab should additionally pay off for patient security and satisfaction. The study may add knowledge how to eliminate stressful exposures and to contribute to more (psychological) security, less output losses and exhaustion during work.

All evaluated guidelines, materials for the psychoeducational intervention in patients and MBSR intervention in practitioners should be transferred in clinical routines.

### Study Status

First participants (patients with elective cardiac catheterization, practitioners/staff at the catheterization lab) for both parts of the randomized, controlled intervention study are recruited and assessed. A pilot study has been completed with 50 participants. Recruitment and implementation are planned for January 2022 until December 2022.

## Ethics Statement

The studies involving human participants were reviewed and approved by Ethics Committee of the University of Potsdam (No. 38/2021). The patients/participants provided their written informed consent to participate in this study.

## Author Contributions

P-MW and KB contributed in study design and initiation. P-MW involved in funding. AB wrote the first manuscript. AB, P-MW, KB, PG, and FB revised the draft and involved in study conduction and management. P-MW and AB provided statistical expertise in clinical trial design. All authors contributed to refinement of the study protocol and approved the final manuscript.

## Funding

This present study was funded by Berufsgenossenschaft fürGesundheitsdienst und Wohlfahrtspflege BGW Deutschland (Nr. 82649910) and by the Deutsche Forschungsgemeinschaft (DFG, German Research Foundation) – Projektnummer 491466077. The funding does not influence data collection, analysis, and interpretation or writing of the manuscript.

## Conflict of Interest

The authors declare that the research was conducted in the absence of any commercial or financial relationships that could be construed as a potential conflict of interest.

## Publisher's Note

All claims expressed in this article are solely those of the authors and do not necessarily represent those of their affiliated organizations, or those of the publisher, the editors and the reviewers. Any product that may be evaluated in this article, or claim that may be made by its manufacturer, is not guaranteed or endorsed by the publisher.

## Footnotes

^1^Approval on 15.10.2021.
